# *Ex vivo* purging of cancer cells from ovarian tissue using photodynamic therapy: a novel strategy to restore fertility in leukemia patients

**DOI:** 10.1093/hropen/hoad005

**Published:** 2023-02-20

**Authors:** Saeid Moghassemi, Arezoo Dadashzadeh, Alessandra Camboni, Olivier Feron, Ricardo Bentes Azevedo, Christiani A Amorim

**Affiliations:** Pôle de Recherche en Physiopathologie de la Reproduction, Institut de Recherche Expérimentale et Clinique, Université Catholique de Louvain, Brussels, Belgium; Pôle de Recherche en Physiopathologie de la Reproduction, Institut de Recherche Expérimentale et Clinique, Université Catholique de Louvain, Brussels, Belgium; Pôle de Recherche en Gynécologie, Institut de Recherche Expérimentale et Clinique, Université Catholique de Louvain, Brussels, Belgium; Service d’Anatomie Pathologique, Cliniques Universitaires Saint-Luc, Brussels, Belgium; Pôle de Pharmacologie et Thérapeutique, Institut de Recherche Expérimentale et Clinique, Université Catholique de Louvain, Brussels, Belgium; Laboratory of Nanobiotechnology, Department of Genetics and Morphology, Institute of Biological Sciences, University of Brasília, Brasília DF, Brazil; Pôle de Recherche en Physiopathologie de la Reproduction, Institut de Recherche Expérimentale et Clinique, Université Catholique de Louvain, Brussels, Belgium

**Keywords:** fertility preservation, photodynamic therapy, nanoparticle, leukemia, ovary, autotransplantation, tissue purging

## Abstract

**STUDY QUESTION:**

Is it possible to purge leukemia cells from ovarian tissue (OT) fragments before transplantation?

**SUMMARY ANSWER:**

Our photodynamic therapy (PDT) approach has been shown to efficiently destroy leukemia cells from tumor-infiltration mimicking models (TIMs), indicating the feasibility of this technique to purge OT samples.

**WHAT IS KNOWN ALREADY:**

Autotransplantation of cryopreserved OT is the most suitable option to preserve fertility for prepubertal girls and women who require immediate cancer treatment. Up until now, more than 200 live births have already been reported after OT cryopreservation and transplantation. Leukemia is the 12th most common cancer in Europe among prepubertal girls and women of reproductive age and in 2020, the estimated number of new leukemia cases was higher than 33 000 in girls between 0 and 19 years old. Unfortunately, once their health has been restored, autotransplantation of cryopreserved OT for leukemia patients is not advised due to the high risk of transferring malignant cells back to the patient leading to leukemia recurrence.

**STUDY DESIGN, SIZE, DURATION:**

To safely transplant the OT from leukemia patients and restore their fertility, our goal was to develop a PDT strategy to eliminate leukemia *ex vivo*. To this end, we designed OR141-loaded niosomes (ORN) to create the most effective formulation for *ex vivo* purging of acute myelogenous leukemia cells from OT fragments (n = 4). Moreover, to ensure that such treatments are not harmful to follicle survival and development so they can be deemed a potential fertility restoration alternative, the effect of the ORN-based PDT purging procedure on follicles was assessed after xenografting the photodynamic-treated OT in SCID mice (n = 5). The work was carried out between September 2020 and April 2022 at the Catholic University of Louvain.

**PARTICIPANTS/MATERIALS, SETTING, METHODS:**

After establishing the best ORN formulation, our PDT approach was used to eradicate HL60 cells from *ex vivo* TIMs prepared by microinjection of a cancer cell suspension into OT fragments. The purging efficiency was analyzed by droplet digital polymerase chain reaction and immunohistochemical analyses. Additionally, we evaluated the effect of ORN-based PDT on follicle density, survival and development, and tissue quality in terms of fibrotic areas and vascularization after 7-day xenotransplantation to immunodeficient mice.

**MAIN RESULTS AND THE ROLE OF CHANCE:**

The *ex vivo* purging of TIMs demonstrated that our PDT strategy could selectively eradicate the malignant cells from tissue fragments without affecting OT normal cells, as evidenced by PCR and immunohistochemical analysis. Regarding the effect of our PDT approach on follicle population and OT quality, our results after xenotransplantation revealed no significant difference between the follicle density of control (non-treated, grafted OT) and PDT-treated groups (2.38 ± 0.63 and 3.21 ± 1.94 morphologically normal follicles/mm^2^, respectively). In addition, our findings showed that the control and PDT-treated OT could be equally vascularized (7.65 ± 1.45% and 9.89 ± 2.21%, respectively). Similarly, the proportions of fibrotic area did not differ between the control (15.96 ± 5.94%) and PDT-treated groups (13.32 ± 3.05%).

**LARGE SCALE DATA:**

N/A.

**LIMITATIONS, REASONS FOR CAUTION:**

This study did not use OT fragments from leukemia patients, but TIMs created after injection of HL60 cells into OT from healthy patients. Therefore, while the results are promising, whether our PDT approach will be equally successful in eliminating malignant cells from leukemia patients remains to be assessed.

**WIDER IMPLICATIONS OF THE FINDINGS:**

Our results showed that the purging procedure causes no significant impairment effect on follicle development and tissue quality, suggesting that our novel PDT procedure could be a promising strategy to destroy leukemia cells in fragments of OT, allowing safe transplantation in cancer survivors.

**STUDY FUNDING/COMPETING INTEREST(S):**

This study was supported by grants from the Fonds National de la Recherche Scientifique de Belgique (FNRS-PDR Convention grant number T.0004.20 awarded to C.A.A.); Fondation Louvain (awarded to C.A.A.; a Ph.D. scholarship awarded to S.M., as part of a legacy from Mr Frans Heyes, and a Ph.D. scholarship awarded to A.D. as part of a legacy from Mrs. Ilse Schirmer); and Foundation Against Cancer (grant number 2018-042 awarded to A.C.). The authors declare no competing interests.

WHAT DOES THIS MEAN FOR PATIENTS? Cancer treatment can damage ovarian function, potentially impairing patient fertility. In prepubertal girls and women who need cancer therapy immediately, it is possible to remove the ovarian tissue before the cancer treatment, cryopreserve it in cryobanks, and transplant it after cancer remission. However, this strategy cannot be offered to leukemia patients because of the high risk of reintroduction of cancer cells present in the cryopreserved ovarian tissue, which could potentially lead to leukemia recurrence. In order to safely transplant the ovarian tissue from these patients, these leukemia cells should be destroyed first. An alternative to selectively eradicate these cells without harming the follicle population could be photodynamic therapy. Based on this hypothesis, this study aimed to develop a novel photosensitizer to destroy leukemia cells in ovarian fragments. Our results showed that we could successfully purge these cells without harming follicle survival and development and ovarian tissue quality. This suggests that our novel procedure could be a promising strategy to destroy leukemia cells in ovary fragments, allowing their safe transplantation in cancer survivors.

## Introduction

Chemo- and radio-therapy, which have led to a high survival rate in cancer patients, may be toxic to the ovary, affecting fertility and endocrine functions. Ovarian tissue (OT) cryopreservation is the most acceptable alternative for preserving fertility for prepubertal girls and women who need immediate cancer treatment. In this strategy, the OT is harvested by laparoscopy and frozen. After patient remission, tissue fragments are thawed and transplanted, restoring endocrine and reproductive functions (Wallace *et al.*, 2005; Maltaris *et al.*, 2007; Donnez and Dolmans, 2017; Dolmans, 2018). Transplantation of cryopreserved OT has been demonstrated to resume ovarian function in more than 90% of the patients, and more than 200 live births have so far been documented using this approach (Dolmans and Donnez, 2020). Unfortunately, however, this strategy cannot be recommended for patients with cancer types with a high risk of metastasis to the ovary due to the significant risk of transmitting malignant cells back to the patient, potentially leading to leukemia recurrence (Amorim, 2016; Dolmans and Amorim, 2019; Dolmans and Donnez, 2020). Among various investigated techniques for restoring fertility in these individuals, including *in vitro* ovarian follicle culture (Telfer and Zelinski, 2013; McLaughlin *et al.*, 2018) and the bioengineered ovary (Amorim and Shikanov, 2016; Chiti *et al.*, 2018; Jafari *et al.*, 2021; Dadashzadeh *et al.*, 2021a,b, 2022), purging the OT fragments of cancerous cells before autotransplantation appears to be the most novel and the most straightforward strategy (Mulder *et al.*, 2019; Eijkenboom *et al.*, 2021; Moghassemi *et al.*, 2021b, 2022b,c,d).

As a promising approach to cancer treatment, photodynamic therapy (PDT) has received significant attention in recent years (Moghassemi *et al.*, 2021a, 2022a,b). Compared with the other common strategies for cancer therapy, PDT has fewer side effects, more selectivity, and can be specifically targeted toward the tumor site (Lehmann, 2007; Chilakamarthi and Giribabu, 2017; Kwiatkowski *et al.*, 2018; Moghassemi *et al.*, 2021a). In this approach, a photosensitizer (PS) accumulates in the target tissue, and once it is activated by light irradiation at a suitable wavelength, significant cytotoxicity occurs in the presence of oxygen (Dougherty *et al.*, 1998; Sharma *et al.*, 2011; Mokwena *et al.*, 2018). OR141 (OR), a novel non-porphyrinic PS, has demonstrated an anticancer immune response in the form of immunogenic cell death induction through oxidation of endoplasmic reticulum-associated proteins after photodynamic activation (Pinto *et al.*, 2016; Doix *et al.*, 2018, 2019; Trempolec *et al.*, 2020; Moghassemi *et al.*, 2022c). To increase the bioavailability, stability, and PDT efficiency of PSs, niosomes have been applied as self-assembled non-ionic surfactant-based vesicular drug delivery systems to encapsulate PSs (Vilela *et al.*, 2022). Niosomes yield not only the advantages mentioned above, but also an increased penetration of PSs into the target tissue (Zheng and Pandey, 2008; Yurt *et al.*, 2017). Of importance, niosomes are also non-immunogenic and biocompatible and can be conjugated with targeting agents (Moghassemi *et al.*, 2017; Yazdi Rouholamini *et al.*, 2018; Dadashzadeh *et al.*, 2020). Despite the high affinity that PSs have to cancer cells (Moghassemi *et al.*, 2021a), their effect on ovarian follicles is unknown.

This research aimed, therefore, to examine the PDT efficiency and selectivity by using an *ex vivo* analysis of tumor-infiltration mimicking models (TIMs). Moreover, the effect of the OR-loaded niosomes (ORN)-based PDT purging procedure on follicle survival and development was assessed after xenografting the photodynamic-treated OT in SCID mice.

## Materials and methods

### Experimental design

**Figure 1.**
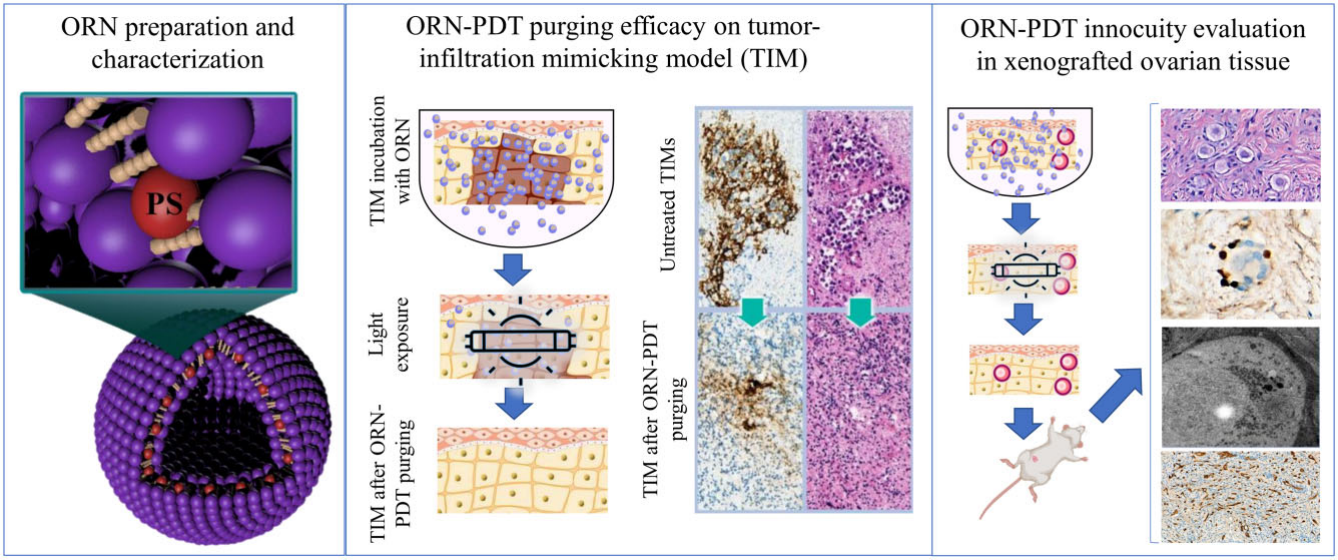
**Experimental design.** (i) Preparation and characterization of OR-141 loaded niosomes (ORN); (ii) assessment of the ORN-photodynamic therapy (PDT) efficacy using tumor models in fragments of ovarian tissue; and (iii) assessment of the PDT specificity using ovarian biopsies of young patients.

This study was divided into three steps: (i) preparation and characterization of ORN; (ii) assessment of the ORN-PDT efficacy using tumor models in fragments of ovarian tissue; and (iii) assessment of the PDT specificity using ovarian biopsies of young patients (Fig. 1). In the first step, the ORN was prepared by our previously optimized method (Moghassemi *et al.*, 2022d), and its characteristics, including size, polydispersity index, and zeta potential were studied. Then, in the following step, the efficiency of the best OR/ORN-PDT purging protocol was analyzed on TIMs created by HL-60 cells microinjected into ovarian tissue. Finally, the third step aimed to evaluate the effect of the PDT approach on the follicle population and ovarian tissue quality to ensure that such treatment is not harmful to follicle survival and development for it to be deemed a potential fertility restoration alternative.

### Ethics

Use of human ovarian tissue was approved by the Institutional Review Board of the Université Catholique de Louvain in May 2019 (IRB references 2012/23MAR/125 and 2018/19DEC/475) and informed consent was obtained from women of reproductive age. Guidelines for animal welfare were approved by the Committee on Animal Research of the Université Catholique de Louvain (reference 2018/UCL/MD/40). Adequate housing and breeding conditions were maintained, as previously reported (Amorim *et al.*, 2012).

### ORN preparation and characterization

ORN was prepared by the reverse-phase evaporation technique, using our optimized and novel formulation (Moghassemi *et al.*, 2022c). Briefly, sorbitan monostearate (S60; S7010; Sigma-Aldrich, Darmstadt, Germany), sorbitan monopalmitate (S40; 388920; Sigma-Aldrich, Darmstadt, Germany), and cholesterol (CH; C8667; Sigma-Aldrich, Darmstadt, Germany), associated with 1 µmol of OR, were mixed in 4 ml of diethyl ether (296082, Sigma-Aldrich, Darmstadt, Germany):dimethyl sulfoxide (DMSO; 482; WAKChemie Medical GmbH, Steinbach, Germany) (10:1 volume ratio). Then, 6 ml of DPBS was added, mixed by vortex mixer at room temperature for 1 min, and sonicated for 10 min in a bath sonicator (Elmasonic S15 H, Elma, Germany) at 10°C. The elimination of organic solvent was performed using a Rotavapor (BUCHI R-3, Flawil, Switzerland) for 30 min and at 60°C, which is above the boiling point of diethyl ether (∼34.6°C) and more than the phase transition temperature of S40 and S60 (∼42°C and ∼53°C, respectively). The optimal suspension underwent high-pressure extrusion passing through two filters with different sizes (0.4 and 0.2 μm) three times, and free OR was removed by two-step ultra-centrifugation (14000×*g*, 4°C, 20 min) and washed with DPBS (14190144; Gibco, Paisley, UK).

### TIM preparation

Frozen-thawed ovarian biopsies (Amorim *et al.*, 2009) were obtained from four postmenopausal multi-organ donors to prepare the TIMs. OT strips were cut into ∼4 × 4 × 2 mm fragments. HL60 cells (98070106; ECACC; Salisbury, UK) were maintained in T75 culture flasks, and after achieving ∼90% confluency, they were washed with 10 ml of medium (RPMI-1640 medium containing 2 mM Glutamine (61870010; Gibco), 10% FBS, and 1% AA). The TIMs were prepared using a protocol from Peek *et al.* (2015). Briefly, a cell suspension with the concentration of 2 × 10^3^ cells/µl was obtained by dilution into a cold (4°C) medium containing Dulbecco’s modified Eagle’s medium (DMEM/GlutaMAX™; 61965026; Gibco) supplemented with 10% heat-inactivated fetal bovine serum (HI FBS; 16140071; Gibco) and 1% antibiotics and antimycotic (AA; 15240-062; Gibco) and injected into the ovarian cortex fragments five times with 5 µl cell suspension per injection, using a sterile needle with a 0.4 mm outer diameter (GONAL-f^®^, Merck Serono, USA). Afterward, the fragments were washed briefly in medium (DMEM/F12 1X (21041-025; Gibco) supplemented with 10% FBS and 1% AA)), cultured in a 6-well plate at 37°C and 5% CO_2_ for 7 days. The medium was renewed every two days.

### Evaluation of the PDT efficacy using TIMs

After 7 days of *in vitro* culture to allow HL60 to proliferate, TIMs were divided into five groups: (i) Control: untreated TIM; (ii) OR-light: TIMs incubated with OR not followed by light exposure; (iii) OR+light: TIMs incubated with OR and then light-exposed (157 mW/cm^2^ day-light LED) for 60 min; (iv) ORN-light: TIMs incubated with ORN, not followed by light exposure; (v) ORN+light: TIMs incubated with ORN and then light-exposed (157 mW/cm^2^ day-light LED) for 60 min.

In the control group, the TIMs were incubated in a culture medium for 24 h at 4°C. In the other four groups, TIMs were incubated with 3 µM OR or ORN for 24 h at 4°C, followed or not by light exposure. All groups were *in vitro* cultured for an additional 5 days before evaluation. The presence of HL60 cells and their proliferation and apoptosis detection in the TIMs were studied by CD43, Ki67, and caspase-3 immunohistochemical stainings, respectively. The TIM preparation and *ex vivo* purging process were performed in quadruplicate.

### Droplet digital polymerase chain reaction analysis

The droplet digital polymerase chain reaction (ddPCR) was used to assess the presence of malignancy in tissue fragments. It quantified the absolute expression of sialophorin (CD43 antigen), a sialoglycoprotein expressed on human neoplastic hematopoietic cell lines, including HL60 (Turzová *et al.*, 1993; Kojima *et al.*, 1994). For this purpose, RNA extraction from paraffin-embedded samples was performed using the RNeasy FFPE kit (73504, Qiagen, Hilden, Germany) according to the manufacturer’s protocol. Then, extracted RNA was quantified with a Qubit™ 4 Fluorometer (ThermoFisher Scientific). For each sample, 75 ng RNA was reverse-transcribed into complementary DNA (cDNA) via the goScript reverse-transcription system (A5000, Promega, Maddison, WI, USA) according to the manufacturer’s instructions. Then, cDNA was mixed with the specific miRNA primers and the fluorescent probe (sialophorin, 4331182, ThermoFisher) and trapped in the droplets by a QX200 droplet generator system. Finally, after transferring them into a 96-well plate, the ddPCR was performed on a QX200 system (Bio-Rad Laboratories Inc., USA), and the outputs were analyzed with QuantaSoft™ software (Bio-Rad Laboratories).

### Analysis of the PDT toxicity in ovarian biopsies

To be deemed a potential fertility restoration alternative, it is vital to prove that our approach has no impairment effect on follicles and ovarian stromal cells. Therefore, to assess the impact of our PDT protocol on preantral follicles and ovarian cells, ovarian biopsies were used. They were collected from five women of reproductive age (20–33 years of age) after obtaining informed consent. All patients underwent laparoscopic surgery for benign gynecological diseases. The tissue fragments from each patient were frozen using our routine procedure (Gallardo *et al.*, 2018) and, after thawing, they were divided into three groups: (i) pre-graft: untreated OT fragments fixed before xenografting; (ii) untreated graft (Unt-graft): OT fragments without exposure to the PDT protocol that were xenografted and fixed; and (iii) ORN-graft: OT fragments incubated with 3 μM ORN for 24 h at 4°C, light-exposed (157 mW/cm^2^ day-light LED) for 60 min, xenografted, and fixed.

An OT fragment from the Unt-graft and another from the ORN-graft group were transplanted per mouse using the protocol previously described (Amorim *et al.*, 2012). The mice were kept in sterile conditions for 1 week and subsequently were euthanized by cervical dislocation. Upon retrieval, each ovarian graft was divided into a larger piece, which was fixed in formalin for histological and immunohistochemical evaluation. A smaller piece from the pre-graft and ORN-graft fragments was also fixed in a 2.5% glutaraldehyde solution for ultrastructural analysis.

We performed a histological assessment for counting and classifying the ovarian follicles (Gallardo *et al.*, 2018) and immunohistochemical stainings, including Ki-67 and Caspase-3, to evaluate cell proliferation and apoptosis, respectively. In addition, the graft vascularization and fibrotic area were assessed by CD31 and Masson’s trichrome staining. Moreover, the follicle ultrastructure was examined under transmission electron microscopy (TEM, Zeiss-EM 109 electron microscope, Germany).

### Transplantation into SCID mice

Five SCID 8-week-old female mice were provided by Charles River (Brussels, Belgium) and were maintained under germ-free conditions. The grafting procedure was carried out as described previously (Amorim *et al.*, 2011). Briefly, the animals were anesthetized by intraperitoneal injection of ketamine (75 mg/kg; Anesketin, Eurovet, Heusden-Zolder, Belgium) and medetomidine (1 mg/kg; Domitor, Pfizer, Cambridge, MA, USA), and buprenorphine (0.1 mg/kg; Temgesic, Schering Plough, Kenilworth, NJ, USA) was administered for analgesia. A ventral midline skin incision was made, and the abdominal wall opened. Two ovarian grafts were stitched to the anterior wall of the peritoneum at the level of the bladder. A control OT fragment (unt-graft) of each patient was placed on the left side and an ORN-treated OT fragment of the same patient was placed on the right side using 6-0 Surgipro^®^ sutures (Covidien, Ireland). The abdominal wall and skin were then closed with absorbable 4-0 Surgipro^®^ sutures. After surgery, anesthesia was reversed by injection of atipamezole (1 mg/kg; Antisedan, Pfizer). After 7 days, the animals were euthanized and the grafts were recovered and fixed in formalin.

### Histologic evaluation

OT fragments, TIMs (with and without treatments) and HL60 embedded in 1% agarose hydrogel were fixed in 4% formaldehyde solution before embedding in paraffin. Sections of 5-µm thickness were cut and placed on Superfrost Plus slides (Menzel-Glaser, Germany). For histological analysis and to select sections for subsequent immunohistochemical staining, every sixth slide was stained with hematoxylin and eosin (Merck, Darmstadt, Germany). Only follicles with a visible nucleus were counted to be sure that no preantral follicles were counted twice. Follicles were then categorized as primordial or growing (intermediate, primary, or secondary) based on granulosa cell shape and the number of layers around the oocyte (Gougeon, 1986). The integrity of the basement membrane, cellular density, the presence or absence of pyknotic structures, and the integrity of the oocyte was used to assess follicular quality. Based on these criteria, follicles were classified as morphologically normal or degenerated, and only normal follicles were characterized and quantified. After providing the hematoxylin–eosin slides, a Pannoramic P250 Flash digital slide scanner (3DHISTECH Ltd., Budapest, Hungary) was used to scan the sections. Counting ovarian follicles in three randomly selected sections of each pre-graft, control, and ORN-treated group were used to assess ovarian follicle density (Amorim *et al.*, 2013).

### Immunohistochemical analysis

To evaluate the presence of HL60 cells, their proliferation, and graft vascularization, sections were stained with CD43, Ki-67, and CD31 using an automated IHC assay on the BenchMark ULTRA (Ventana Medical Systems Inc., USA). The proportion of vessel area was calculated using ImageJ software (version 1.53e, Wayne Rasband, NIH, USA). Additionally, rabbit anti-cleaved caspase-3 was employed for apoptosis detection. After deparaffinization by Histosafe (Yvsolab SA, Beerse, Belgium) and rehydration by isopropanol (Merck, Darmstadt, Germany) and water, endogenous peroxidase activity was inhibited by 30 min incubation with 0.3% H_2_O_2_ in demineralized water at room temperature. Then, the sections were demasked in citrate buffer and Triton X-100 (pH 6) for 75 min in a water bath at 98°C, and non-specific binding sites were blocked by incubation with normal bovine serum albumin (1%) for 30 min. The sections were then incubated overnight at 4°C with the primary antibody. Table I lists the antibodies, incubation conditions, and positive and negative control tissues. The dilution solution without any primary antibody was provided as a negative control. The slides were subsequently incubated for 60 min at RT with an anti-rabbit secondary antibody. After 15 min incubation with chromogen-substrate (diaminobenzidine, DAB), Mayer’s hematoxylin was used as a counterstain. The slides were digitized using a Pannoramic P250 Flash digital slide scanner (3DHISTECH Ltd.).

**Table I:** Summary of immunohistochemical protocols used to evaluate the survival, development, and function of follicles and graft vascularization.

Stainings	Antibody type	Dilution	Control	Ref
Ki67	Monoclonal	1:90 in TBS +1% NGS+0.1% BSA	Proliferative endometrium	M7240; Dako, Glostrup, Denmark
CD31	Monoclonal	1:100 in TBS +1% NGS+0.1% BSA	Kidney	AB56299; Abcam, Cambridge, UK
CD43	Monoclonal	1:150 in TBS +1% NGS+0.1% BSA	Tonsil	1-CD160; Quartett GmbH, Berlin, Germany
Caspase-3	Polyclonal (rabbit)	1:200 in TBS + 4% NGS+0.4% BSA	Human tonsil	9661S; Cell Signaling Technology, Beverly, MA, USA
GDF-9	Monoclonal	1:6000 in TBS + 2.5% BSA +1% non-fat milk	Testis	AB-325-AG012; Anshlabs, Webster, TX, USA

GDF-9, growth differentiation factor-9; BSA, bovine serum albumin; NGS, normal goat serum; TBS, tris-buffered saline.

Proliferative follicles were defined as those with at least one granulosa cell stained positive for Ki67. Follicles were classified as positive for caspase-3 immunostaining if more than 50% of granulosa cells and/or the oocyte were positive.

### Ultrastructure analysis

After fixation, the tissue samples were rinsed in phosphate-buffered saline solution and postfixed in 1% osmium tetroxide, dehydrated through an ascending series of ethanol, immersed in propylene oxide, and embedded in Epon 812 (Agar Scientific, Essex, UK). Afterwards, ultrathin (60–90 nm) sections were obtained using a Leica EM UC7 ultramicrotome (Wetzlar, Germany) mounted on copper grids, contrasted with saturated uranyl acetate and lead citrate, and examined by transmission electron microscopy (TEM, Zeiss-EM 109 electron microscope, Germany). The appearance, organelles distribution, membranes integrity, and connections between the granulosa cells and the oocyte were investigated.

### Fibrosis

The tissue sections from the extremities and middle of the fragment were used to measure the relative surface area of fibrosis. Fibrotic regions were characterized by poor cellularity, as demonstrated by a lower number of cell nuclei and collagen deposition (Dath *et al.*, 2010; Amorim *et al.*, 2012). Tissue that has been replaced by collagenous connective tissue appears blue in Masson’s trichrome staining, making fibrotic regions identifiable. Sections were scanned by a Pannoramic P250 Flash digital slide scanner (3DHISTECH Ltd.), and measurement of fibrotic areas and total section areas was carried out using the Caseviewer 2.4 program (3DHISTECH Ltd.).

### Statistical analysis

The quantitative data were reported as mean±SD, and statistical analysis of the data was carried out using one-way ANOVA. *P*-values <0.05 were considered statistically significant. In graphs, the error bars represent a single sample standard deviation. Unpaired *t*-tests were used to compare individual conditions, with Bonferroni multiple testing correction for multiple related tests where appropriate.

## Results

### ORN characterization

ORN was prepared in dark and sterile conditions based on established optimal formulation obtained by response surface methodology modeling to receive the best DLS characteristics and highest entrapment efficiency (Table II). The polydispersity index of nanoparticles prepared by optimized formulation was under 0.2, which demonstrates a sharp size distribution and low tendency to aggregate. The ORN zeta potential around −25 mV shows high electrostatic stabilization, so nanovesicles can be perfectly suspended in an aqueous solution. This is crucial for their storage and administration (Bayindir and Yuksel, 2010; Moghassemi and Hadjizadeh, 2014).

**Table II:** ORN dynamic light scattering characterization (zeta potential, size, and polydispersity index), and the OR-141 entrapment efficiency.

Design data	Zeta potential	Size (nm)	Polydispersity index	Entrapment efficiency (%)
Mean±SD	−24.20 ± 1.06	301.45 ± 1.95	0.19 ± 0.01	71.31 ± 4.39

ORN, OR-141 loaded niosomes.

### Ex vivo PDT purging of TIMs

**Figure 2.**
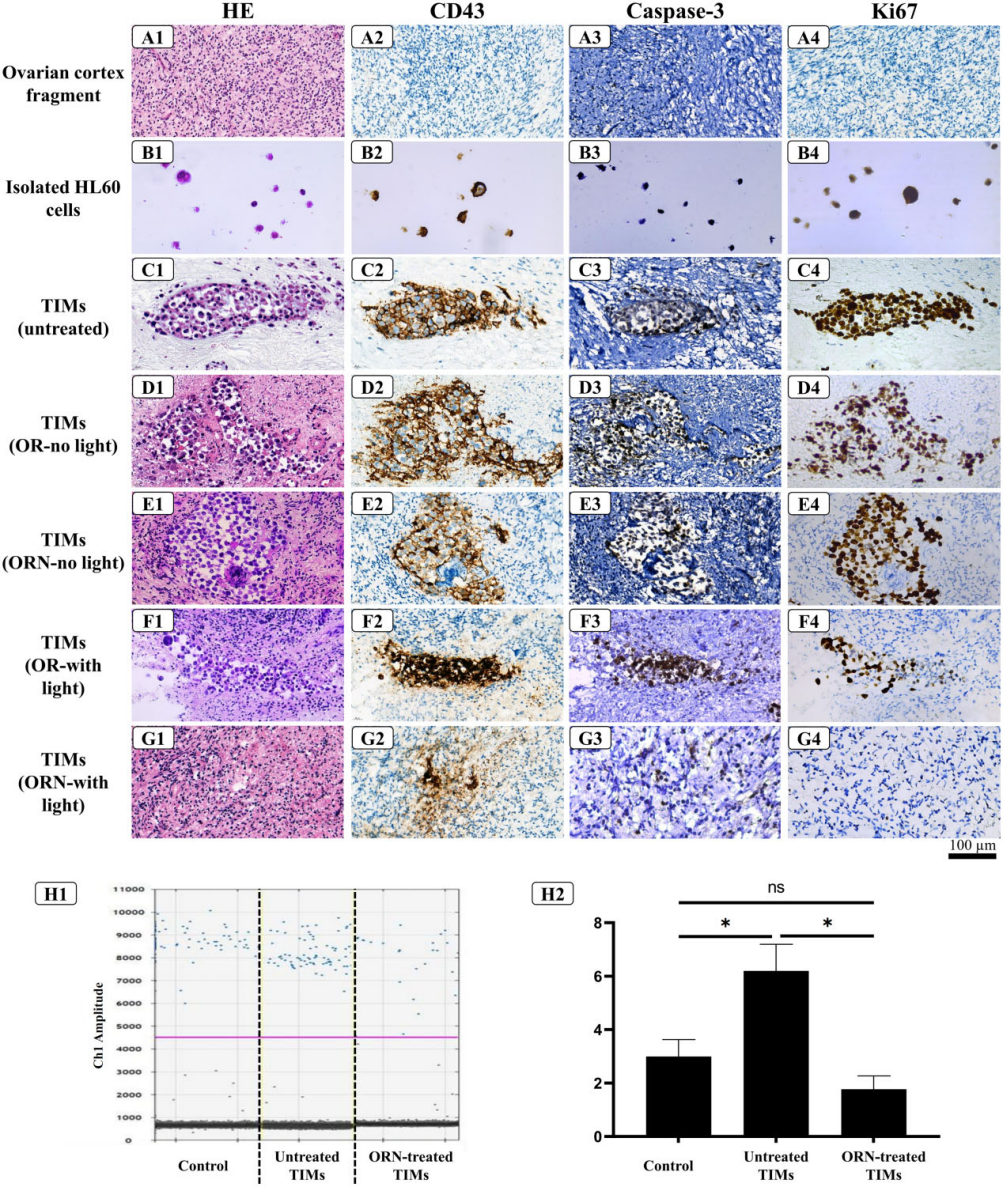
**Assessment of the tumor-infiltration mimicking model (TIM).** Histological and immunohistochemical (CD43, Caspase-3 and Ki67) analyses of ovarian tissue (OT) (**A**), HL60 cells embedded in 1% agarose (**B**), tumor formation by HL60 cells in human ovarian cortex tissue (**C**), and leukemic tumor eradication efficiency in TIMs by photo-dynamic therapy (PDT) purging with OR-141 (OR)/OR-loaded niosomes (ORN) with or without the light exposure (**D**–**G**). In the light-exposed OR-treated TIM, the HL60 cells display characteristics of apoptosis, but there are still some Ki67-positive cancer cells after post-PDT incubation time. In the light-exposed ORN-treated TIM, the HL60 cells were disintegrating, as evidenced by the diffuse brownish area in the CD43 staining due to the HL60 cell debris and the lack of Ki67 protein expression (×400 magnification). This was confirmed by the detection of sialophorin gene amplification in ovarian tissues (OTs) (negative control), TIMs (positive control) and ORN-treated TIMs (**H1**), and droplet-digital (dd)PCR quantification (**H2**) (*P < 0.01) (n = 4).

Upon incubation of HL60 cells with OT fragments, we successfully created the TIMs, as evidenced by densely packed tumor masses containing proliferative leukemia cells in control tissue fragments (Fig. 2C1, C2, and C4). CD43 staining of HL60 cells revealed close contact between the tumor cells and OT (Fig. 2C2, D2, and E2). The (immuno-) histochemical staining of our heterotypic cell models treated by OR or ORN demonstrated that cancer cells were not sensitive to the PSs when they were not exposed to light (Fig. 2D1–4 and E1–4). In the light-exposed OR-treated group, the PDT procedure induced apoptosis (Fig. 2F3), but 5 days post-PDT, there were still some signs of proliferation in the tumor sites, as showed by Ki67-positive HL60 cells (Fig. 2F2 and F4). By contrast, in the ORN-treated light-exposed group, no Ki67-positive HL60 cells were detectable after PDT (Fig. 2G4). In addition, CD43 and caspase-3 staining showed the presence of leukemia cell debris after ORN-based PDT (Fig. 2G2 and G3). In addition, CD43 gene expression confirmed the reduced number of malignant cells in the ORN-treated TIM, where sialophorin-detected gene levels were not above the background level and were significantly lower than in the untreated TIM (Fig. 2H1 and H2).

### PDT innocuity to preantral follicles and ovarian tissue

#### Follicle survival and development

**Figure 3.**
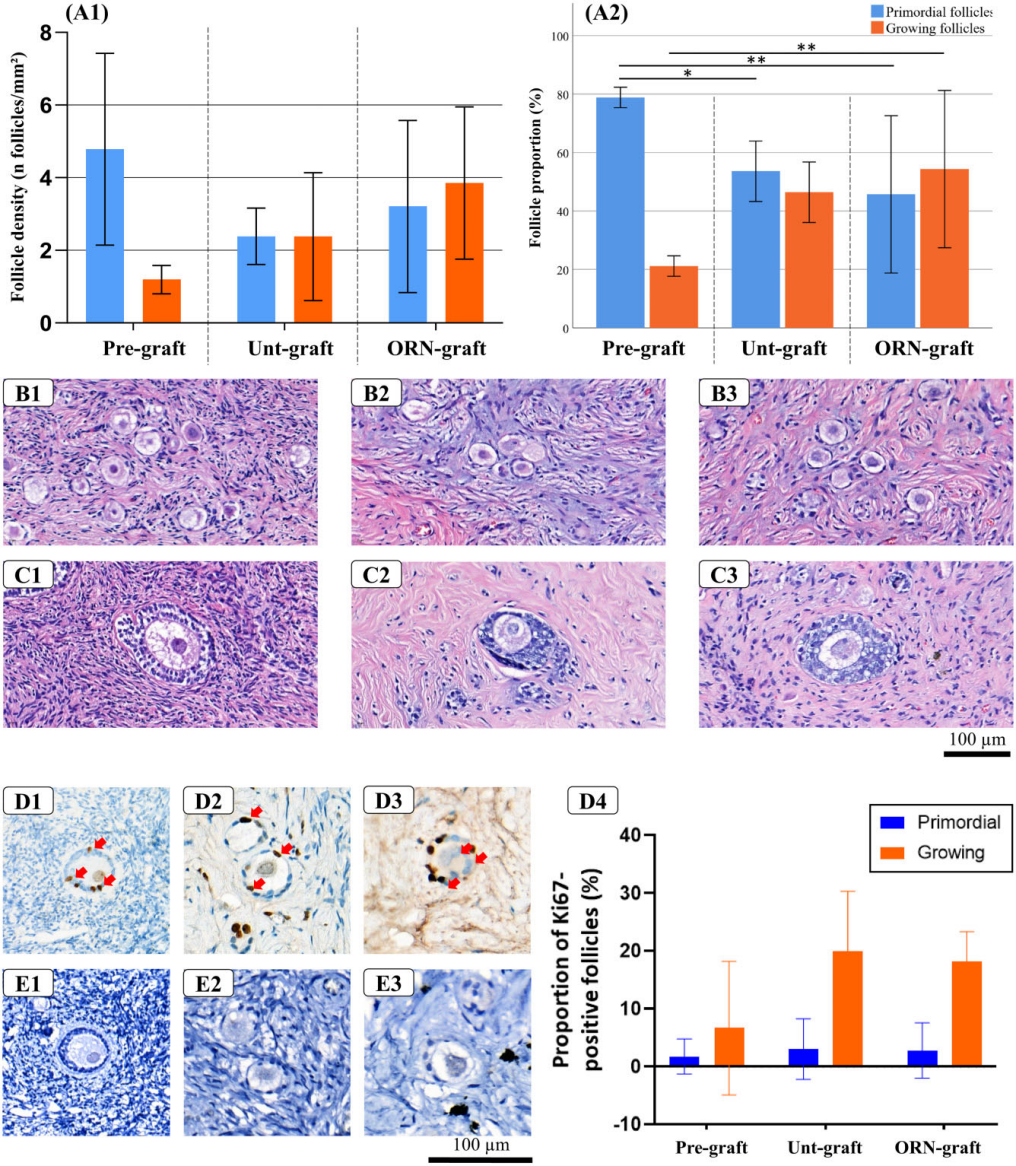
**Analysis of the follicle population before and after xenografting.** Follicle density (**A1**) and proportion (**A2**) in pre-graft, untreated (unt)-graft, and OR-141-loaded niosomes (ORN)-graft ovarian tissue (OT) fragments. Primordial and growing follicles from pre-graft (**B1, C1**), unt-graft (**B2, C2**), and ORN-graft groups (**B3, C3**). Ki67-positive granulosa cells (brown stain) in preantral follicles in pre-graft (**D1**), unt-graft (**D2**), and ORN-graft OT (**D3**) fragments and proportion of Ki67-positive follicles in the three different groups (**D4**). Lack of caspase-3 staining in pre-graft (**E1**), unt-graft (**E2**), and ORN-graft OT (**E3**) samples (n = 5).

Follicle density in three randomly chosen sections from pre-graft, unt-graft, and ORN-graft OT fragments ranged from 4.76 ± 2.48 to 7.06 ± 3.92 follicles/mm^2^ (Table III). The unt-graft and ORN-graft fragments had no significant difference in their density and proportion of follicles (Fig. 3A1 and A2). Morphologically normal primordial and growing (primary and secondary) follicles, with spherical oocytes surrounded by granulosa cells in intimate contact with each other and covered by a visible basal membrane, were observed in both unt-graft and ORN-graft groups (Fig. 3B1–3 and C1–3).

**Table III:** Follicle density (mean ± SD) for different groups.

Groups	Morphologically normal follicles			
	Primordial	Primary	Secondary	Antral
Pre-graft	4.78 ± 2.15	1.10 ± 0.38	0.04 ± 0.05	0.05 ± 0.07
Unt-graft	2.38 ± 0.63	2.14 ± 1.63	0.24 ± 0.22	–
ORN-graft	3.21 ± 1.94	3.41 ± 1.61	0.44 ± 0.36	–

ORN, OR-141 loaded niosomes; Pre-graft, untreated ovary fragments before xenotransplantation; Unt-graft, untreated ovary fragments after xenotransplantation; ORN-graft, ORN-treated ovary fragments after xenotransplantation.

The proportion of primordial follicles were significantly lower in both unt-graft and ORN-graft groups compared with pre-graft fragments (*P* < 0.01), and the proportion of growing follicles in the ORN-graft group was significantly higher than in the OT fragment before xenografting (*P* < 0.001) (Fig. 3A2). Follicle development in both groups was confirmed by Ki67-positive granulosa cells (Fig. 3D4). Interestingly, none of the follicles after xenografting were positive for caspase-3 (Fig. 3E1–3).

#### Ultrastructure analysis of the follicles

A total of 44 preantral follicles were analyzed: 22 in the pre-graft group and 22 in the ORN-graft group. In the frozen-thawed pre-graft group, the majority of follicles were at the primordial stage (13/22, 59%), while in the ORN grafted group, only 36% of follicles (8/22) were at the primordial stage and 64% (14/22) were at the early growing stage (Table IV).

**Table IV:** Classification of preantral follicles based on the ultrastructure study on pre-graft and ORN-graft groups.

Groups	Follicles	Primordial	Early growing	Healthy looking	Altered
Pre-graft	22	59% (13/22)	41% (9/22)	72% (16/22)	28% (6/22)
ORN-graft	22	36% (8/22)	64% (14/22)	68% (15/22)	32% (7/22)

ORN, OR-141 loaded niosomes; Pre-graft, untreated ovary fragments before xenotransplantation; ORN-graft, ORN-treated ovary fragments after xenotransplantation.

**Figure 4.**
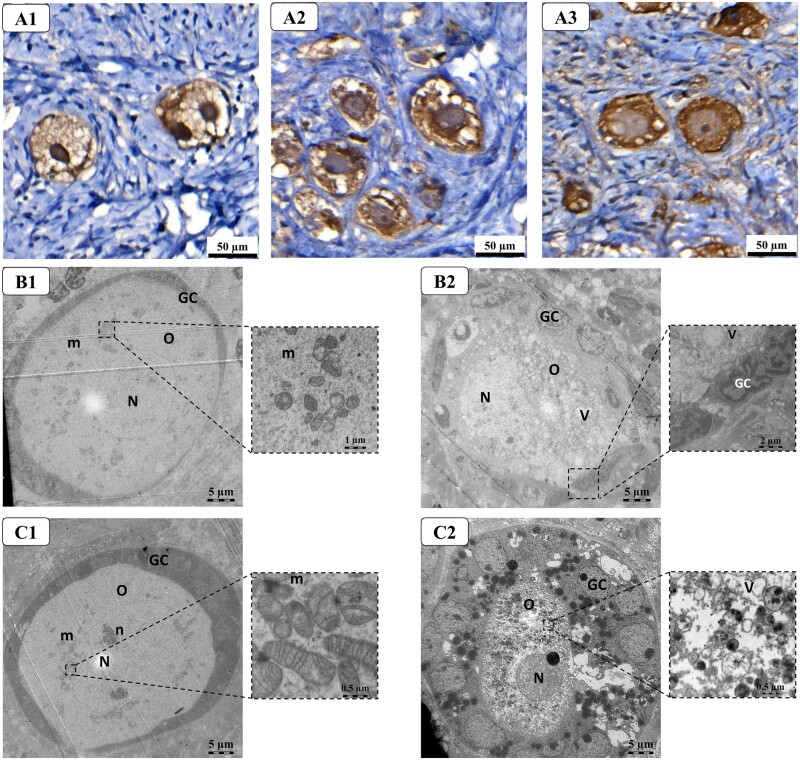
**Follicle analysis.** Microscopic analysis of preantral follicles: healthy-looking (**A1**) and altered (**A2, A3**). Ultrastructure of preantral follicles from pre-graft groups: healthy-looking (**B1**) and altered (**B2**). Ultrastructure of preantral follicles OR-141-loaded niosomes (ORN)-graft groups: healthy-looking (**C1**) and altered (**C2**) (n = 5) scale bar = 5 µm; GC, granulosa cell; N, oocyte nucleus; m, mitochondria; O, oocyte; V, vacuoles.

At the ultrastructural level, 72% and 68% of follicles belonging to the pre-graft group and the ORN-graft group, respectively, showed well-preserved oocyte and follicular compartments (Table IV, Fig. 4A1). The oocytes showed a central nucleus with dispersed chromatin and one to two nucleoli. In the oocyte cytoplasm, numerous mitochondria with arciform and peripheral cristae, Golgi cisternae, rough and smooth endoplasmic reticulum (RER and SER, respectively), lysosomes, and a few multi-vesicular bodies were observed mainly around the nucleus forming the Balbiani body (Fig. 4B1, inset and Fig. 4C1, inset). In all these follicles, the connection between oocyte microvilli and follicular cell interdigitations was well maintained. Follicular cells, arranged in one layer surrounding the oocyte, exhibited an irregular nucleus with chromatin condensed on the nuclear envelope. Scattered organelles, such as mitochondria with transverse cristae, RER, SER, and lysosomes, were present in the cytoplasm of follicular cells.

Altered follicles were observed in both pre-graft (28%) and ORN-graft groups (32%). In pre-grafted tissue, altered follicles showed an oocyte with an irregular nuclear envelope and swollen cytoplasmic organelles (Fig. 4B2). Some follicular cells presented condensation of the chromatin and cytoplasm (Fig. 4B2, inset). In the ORN grafted group, different alterations were observed in follicular and oocyte compartments. Follicular cells showed numerous lipid droplets in their cytoplasm and some cellular detachments between them (Fig. 4C2). Connections with oocytes were always maintained. The oocytes were characterized by the presence of condensed chromatin in the nucleus and a large number of vacuoles in their cytoplasm (Fig. 4C2, inset).

#### Vascularization and fibrosis

**Figure 5.**
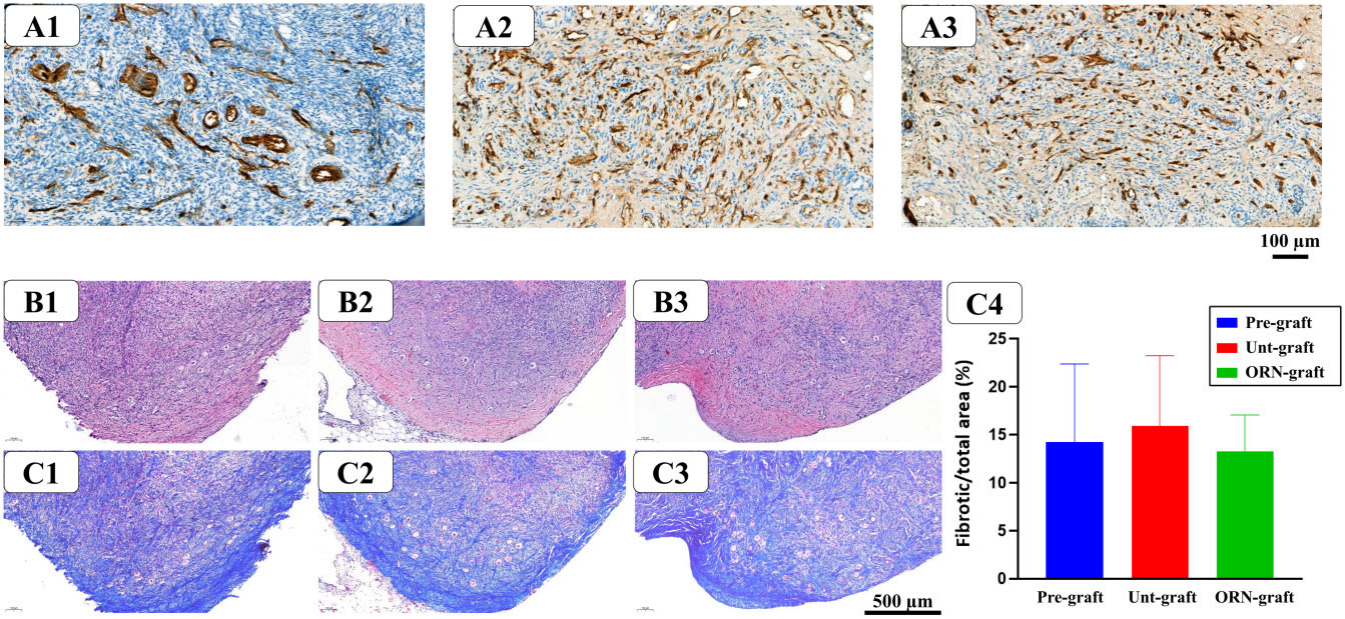
**Ovarian tissue (OT) analysis.** CD31 immunostaining: positive staining (in brown) in pre-graft (**A1**), untreated (unt)-graft (**A2**), and OR-141-loaded niosomes (ORN)-graft groups (**A3**); (n = 5) scale bar = 100 µm. OT stained with hematoxylin–eosin from pre-graft (**B1**), unt-graft (**B2**), and ORN-graft (**B3**) OT groups and fibrotic areas evidenced by Masson’s trichrome staining (**C1–3**) and their proportions in the three different groups (**C4**) (n = 5).

Vascularization was detected in OT after 1 week of xenografting and quantified by evaluating the proportion of CD31-immunostained section areas. Vessel sections area did not differ between the unt-graft (7.65 ± 1.45%) and ORN-graft group (9.89 ± 2.21) (Fig. 5A2, and A3), demonstrating that our PDT approach does not affect endothelial cells or angiogenesis. The proportion of fibrotic area was also similar among groups (pre-graft: 14.22 ± 6.65%; unt-graft: 15.96 ± 5.94%, and ORN-graft: 13.32 ± 3.05%), which indicates that our treatment does not have a deleterious effect on stromal cells (Fig. 5C1–4).

## Discussion

In our study, TIMs were used to replace OT fragments from leukemia patients because the latter are very precious and limited for research applications. Moreover, since we cannot ensure that all tissue samples from these patients contain malignant cells, it is not possible to correctly evaluate the efficacy of our purging protocol (Eijkenboom *et al.*, 2021). It is also important to highlight that our choice to carry out the ORN incubation for 24 h at 4°C was to benefit from the period of OT transport if our PDT strategy is to be implemented in clinical practice (Vilela *et al.*, 2021).

The *ex vivo* purging from TIMs showed that the niosomal formulation could eradicate the cancer cells without affecting ovarian stromal cells, confirming the findings from our *in vitro* study. Based on our findings, we can suggest that the niosomal formulation improved penetration efficiency and purging, as ORN completely eradicated the HL60 cells, while some proliferative leukemia cells could still be found in the OR group. Indeed, the niosomal formulation has been shown to enhance tissue penetration and bioavailability in *ex vivo* studies. Bragagni *et al.* (2015) reported that niosomal 5-aminolevulinic acid yielded a more effective penetration in human skin samples. Compared with other purging procedures using a combination treatment of 3.0 μM verteporfin and 80 μM Imatinib (Mulder *et al.*, 2019), which is based on the inhibition of yes-associated protein and transcriptional co-activator with PDZ-binding motif oncoproteins, ORN-PDT *ex vivo* purging is based on phototoxicity and appears easier and safer. Furthermore, the PDT-based purging technique has light-dependent toxicity. It is safer than chemotherapy-based purging since, even if the PS remains in the tissue, it does not cause any undesired toxicity in the body after transplantation.

While the *in vitro* and *ex vivo* studies demonstrate the successful effect of our ORN-based PDT strategy on malignant cells, our xenografting experiments demonstrate that it does not affect follicle survival and early development. Indeed, there was no difference in the follicle density, and proportion between unt-graft and ORN-graft groups. Moreover, the follicle ultrastructure from both groups was comparable with that in previous studies on follicles from grafted OTs (Borges *et al.*, 2009; Gallardo *et al.*, 2018). Similarly, fibrotic areas and vascularization were similar in the two groups, showing that the ORN-based PDT procedure had no significant detrimental impact on tissue quality.

In conclusion, our PDT strategy with the ORN formulation showed a high potential to purge leukemia cells from OT fragments, by limiting the unwanted effects of OR on stromal cells and allowing selective eradication of cancer cells in OT fragments. Moreover, ORNs are also a promising strategy for *ex vivo* PDT-based purging of OT because they do not lead to adverse effects on preantral follicles and OT. To confirm our promising results, our follow up study will focus on applying our PDT strategy to the OT of leukemia patients, with long-term xenografting to assess the eradication of cancer cells, complete follicle development and oocyte quality.

## Data Availability

The data underlying this article will be shared on reasonable request to the corresponding author.
